# Ketogenic Diet in the Treatment of Malignant Gliomas: A Systematic Review

**DOI:** 10.3390/nu18132166

**Published:** 2026-07-03

**Authors:** Michela Persiani, Laura Dallolio, Alice Masini, Yari Longobucco, Simona Bertoli, Emilia Guberti, Marta Pellizzari, Maria Letizia Petroni, Federica Valeriani, Francesca Gallè, Rossella Sacchetti

**Affiliations:** 1 Department of Quality of Life, University of Bologna, 40126 Bologna, Italy; michela.persiani3@unibo.it; 2 Department of Biomedical and Neuromotor Sciences DIBINEM, University of Bologna, 40126 Bologna, Italy; laura.dallolio@unibo.it; 3 Department of Translational Medicine, University of Eastern Piedmont, 28100 Novara, Italy; 4 Department of Health Sciences, University of Florence, 50121 Florence, Italy; yari.longobucco@unifi.it; 5 Department of Food, Environmental and Nutritional Sciences, University of Milan, 20122 Milan, Italy; simona.bertoli@unimi.it; 6 Istituto Auxologico Italiano IRCCS, Obesity Unit and Laboratory of Nutrition and Obesity Research, Department of Endocrine and Metabolic Diseases, 20145 Milan, Italy; marta.pellizzari@unimi.it; 7 Board Food and Nutrition Società Italiana di Igiene e Medicina Preventiva, 40126 Bologna, Italy; emilia.guberti@gmail.com; 8 Department of Medical and Surgical Sciences, University of Bologna, 40126 Bologna, Italy; marialetizia.petroni@unibo.it; 9 Department of Movement, Human, and Health Sciences, University of Rome Foro Italico, 00135 Rome, Italy; federica.valeriani@uniroma4.it; 10 Department of Medical, Movement and Wellbeing Sciences, University of Naples “Parthenope”, 80133 Naples, Italy; francesca.galle@uniparthenope.it; 11 Department of Education Studies “Giovanni Maria Bertin”, University of Bologna, 40126 Bologna, Italy; rossella.sacchetti@unibo.it

**Keywords:** glioma, ketogenic diet, safety, tolerability, overall survival, quality of life

## Abstract

**Background:** The Ketogenic Diet (KD) has been proposed as an adjunct to standard therapy for gliomas by targeting tumor glycolysis. We systematically reviewed evidence on KD interventions in patients with glioma across multiple outcomes. **Methods:** Following Cochrane guidelines, we searched MEDLINE, Embase, and Cochrane Library (until 1 February 2025) for studies on humans with glioma treated with any KD versus usual care or other diets. Outcomes included overall survival (OS) and progression-free survival (PFS), quality of life (QoL), biochemical markers (glucose, ketones, glucose–ketone index GKI), anthropometric measures, safety, and tolerability. All study designs were eligible. The quality of the studies was assessed with JBI tools. Data were narratively synthesized due to heterogeneity. The PROSPERO registration is CRD42024547388. **Results:** Twenty-three studies (306 patients) were included: two RCTs, 11 quasi-experimental studies, six case series, and four case reports. Dietary interventions included classic KD, modified KD, medium-chain triglyceride KD, and Modified Atkins Diet, sometimes combined with fasting. The overall dropout was 20.3%, mainly due to dietary restrictiveness, disease progression, adverse effects, or QoL concerns. Survival results were inconsistent: RCTs showed no significant OS/PFS differences versus controls, although exploratory analyses suggested better outcomes with lower glucose levels. Ketosis was commonly achieved; glucose reductions were variable, and GKI was rarely reported, with target values seldom reached outside fasting periods. Weight/BMI generally decreased modestly or remained stable; adverse events were mostly mild and tolerability was acceptable. **Conclusions:** Evidence for KDs in glioma patients is heterogeneous and limited. Although ketosis and safety are achievable, survival or QoL benefits remain unproven. Given the methodological challenges, the clinical complexity, and the promising potential of the Ketogenic Diet, further well-designed studies are needed to clarify its clinical utility in glioma treatment.

## 1. Introduction

Gliomas usually originate from glial stem or progenitor cells and can involve both the brain and the spinal cord. The 2021 *WHO Classification of Tumors of the Central Nervous System* (5th edition; WHO CNS 5) recognizes four principal families of gliomas: adult-type diffuse gliomas; pediatric-type diffuse low-grade gliomas; pediatric-type diffuse high-grade gliomas; and circumscribed astrocytic gliomas [1].

According to the Central Brain Tumor Registry of the United States (CBTRUS), astrocytic tumors—including glioblastoma—represent approximately 77.5% of all gliomas, with glioblastoma alone accounting for 58.4% of malignant cases. Overall, gliomas constitute about 24.5% of all primary brain tumors and 80.9% of malignant intracranial neoplasms in adults. The incidence in adults is estimated at 5.51 per 100,000 males and 3.65 per 100,000 females, with glioblastoma peaking at 3.23 per 100,000, whereas lower-grade astrocytomas and oligodendrogliomas occur at 0.11–0.46 per 100,000 [2].

In 2016, the WHO incorporated isocitrate dehydrogenase (IDH) mutation status into CNS tumor classification, recognizing it as the strongest prognostic marker: IDH-mutant gliomas show epigenetic silencing of glycolytic genes, slower growth, and markedly longer overall and progression-free survival than IDH-wildtype tumors [3,4]. O6-methylguanine-DNA methyltransferase (MGMT) promoter methylation further impairs DNA repair after alkylating therapy, increasing responsiveness to temozolomide [5,6,7,8,9]. The combined IDH-mutant/MGMT-methylated subtype confers the most favorable five-year survival, highlighting the importance of integrated molecular diagnostics in glioma management [6,9,10].

The metabolism of tumor cells shows high rates of aerobic glycolysis, a phenomenon known as “Warburg effect” [11].

When glucose is not available, human cells can use ketone bodies, produced through fatty acid oxidation in the liver, as an energy source, which can overcome the blood–brain barrier and substitute glucose [12]. Instead, cancer cells mainly depend on glucose for their growth and survival [13].

Therefore, over the past decades, increasing understanding of the metabolic features of cancer cells has driven research toward calorie-restricted nutritional interventions capable of limiting tumor growth and proliferation. Dietary strategies designed to modulate glycemic levels and induce systemic ketosis—such as energy-restricted diets or intermittent fasting—have, therefore, been explored in patients with CNS tumors [14,15]. In particular, interest in the use of the KD in cancer treatment has grown [16]. KD is defined as a restrictive therapeutic diet model that is generally hyperlipidic, normoproteic, and hypoglucidic [11,17,18]. Several dietary regimens are collectively referred to as ketogenic, based on their macronutrient composition. The most established is the Classic Ketogenic Diet (CKD), typically providing a fat-to-protein-plus-carbohydrate ratio of 3–4:1. More flexible and palatable variants, such as the Medium-Chain Triglyceride Ketogenic Diet (MCT-KD), the Modified Atkins Diet (MAD) and the Modified Ketogenic Diet (MKD) have also been implemented in clinical practice. The Very Low-Calorie Ketogenic Diet (VLCKD) represents a more restrictive approach and induces rapid weight loss during the short ketose phase (12–16 weeks); however, it has not yet been reported in the therapeutic management of brain tumors but only in people living with obesity [16,19,20].

By depriving tumor cells of their main energy fuel, KDs can offer a therapeutic approach targeting the Warburg effect in glycolytic tumors such as malignant gliomas [14].

Currently, KDs have been successfully introduced in the therapy of several neurological and non-neurological diseases, as well as cancer [21]. Clinical studies analyzing the therapeutic role of KDs in central nervous system tumors, especially in gliomas, have increased in recent years [22].

However, recent evidence shows that glioma cells can also utilize other substrates than glucose, such as amino acids and fatty acids, to obtain raw materials and energy for cellular maintenance and tasks [23,24]. In their experiments on mouse models, Sperry et al. found that the administration of a KD led to a compensatory fatty acid oxidation which, combined with ketone body metabolism, was sufficient to sustain glioblastoma growth and decreased overall survival [25]. In addition, different adaptation mechanisms have been observed in different types of brain tumors [26]. Some types of cancer cells possess the ketolytic enzymes required to generate acetyl Co-A from ketone bodies, feeding the citric acid cycle that can be used to generate ATP. Therefore, nutritional changes may be effective in some patients and ineffective in others.

Given this inconsistency, the aim of this systematic review is to provide a comprehensive picture of studies published to date on the role of KDs as an adjuvant treatment of gliomas.

We reviewed the available scientific evidence regarding the effects of various KD interventions on survival, quality of life, and diet-related metabolic and anthropometric parameters in patients with glioma. We also analyzed the safety and tolerability of KDs, as well as the reasons for treatment discontinuation and patient dropout rate.

## 2. Materials and Methods

The present systematic review was performed according to the Cochrane systematic review of interventions’ methodologies [27] and registered in PROSPERO CRD42024547388.

### 2.1. Search Strategy and Study Selection

A comprehensive search strategy was developed to identify studies of interest published until May 2024. The search was subsequently updated on 1 February 2025. Articles were searched in Medline, Embase, and the Cochrane Library using the following search terms: “Glioma”, “Diet, Ketogenic”, “Keto-diet*”, “Ketogen*”, “Keto*”, “Low carb*”, and “High-fat”; these terms were combined using the Boolean operators “AND” and “OR”. Full search strategies for each database are provided in Supplemental Table S1. The search terms were adapted for each specific database.

All studies identified during the first search were uploaded into Zotero and duplicates were removed. Additionally, the bibliographic references of the articles considered eligible for inclusion were also screened, whenever the title appeared to be related to the search term.

### 2.2. Eligibility Criteria

The systematic literature review started with a formulation of the PICOS question [28] (Table 1). We were interested in human patients of any age affected by malignant glioma (P) who were: treated with all types of KDs (including those combined with intermittent fasting), concomitant with standard therapies or not (I); compared with patients following other dietary regimens (C); and to patient dropout, survival, quality of life, biochemical markers (glucose, ketones, GKI, anthropometric measures (weight, BMI), and safety and tolerability as outcomes (O). All study designs were included (e.g., Randomized Controlled Trials, Quasi-Experimental studies, Clinical Trials, Case–Control studies, Case Series, Case Reports) (S).

The exclusion criteria were as follows: (1) publications, such as reviews, study protocols, letters to editors, guidelines, abstracts, book chapters, gray literature; (2) studies with patients affected by different tumors; (3) studies focusing on different dietary regimens; and (4) studies not assessing human subjects.

### 2.3. Data Extraction and Synthesis

Titles and abstracts were independently screened by two reviewers (FV, FG), with full-text assessment for all potentially eligible studies and arbitration by a third reviewer (LD) when needed. Data extraction was performed independently by RS and MP using a standardized Excel form and verified through comparison and consensus. Extracted variables included study characteristics, participant demographics, tumor type, KD regimen and duration, dropout data, survival, quality of life, metabolic and anthropometric outcomes, tolerability and safety.

Ketogenic interventions were grouped into four categories (Classic KD, Medium-Chain Triglyceride KD, Modified Atkins Diet, Modified KD), with detailed protocol features listed in Supplemental Table S2. Given the heterogeneity across study designs and reported outcomes, data were synthesized narratively and descriptively using means or medians and corresponding variability measures as reported in the original studies.

### 2.4. Methodological Quality Assessment

Methodological quality was performed using the Joanna Briggs Institute (JBI) Critical Appraisal Checklists [29].

Six authors (AM, FG, FV, YL, MP, RS) independently assessed the methodological quality of each included study, applying the appropriate JBI critical appraisal checklist.

Any disagreements in appraisal were resolved by consensus, with another reviewer consulted if necessary.

The JBI checklist score was as follows: “Yes” with 1 point, “No” and “Unclear” with 0 points. Scores were given for adherence to each of those aspects, ranging from 0 to 13 for RCT, from 0 to 9 for quasi-experimental study, from 0 to 10 for case series and finally from 0 to 8 for case report [30]. The sum of the points was classified as the percentage of the items present, considering the recommendations of the authors Camp and Legge [31]. Thus, a score lower than 70% was classified as low quality, between 70 and 79% of the checklist criteria was classified as medium–high quality, between 80 and 90% was assigned high quality, and a score greater than 90% of the criteria was classified as excellent quality.

## 3. Results

### 3.1. Study Selection and Data Synthesis

**Figure 1:**
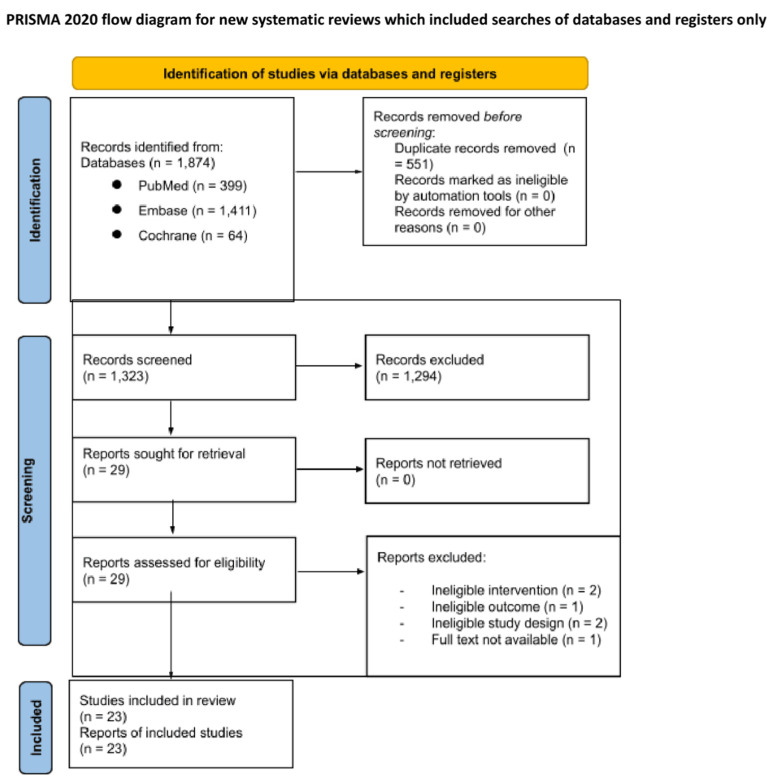
Prisma flow chart [32].

A total of 1874 records were identified across MEDLINE, EMBASE, and the Cochrane Library, with 551 duplicates removed. After screening 1323 titles and abstracts, 29 full texts were assessed. Twenty-three studies met the inclusion criteria (Figure 1; Supplemental Table S3).

Due to methodological heterogeneity, a meta-analysis of the results of selected studies was not possible. Results were, therefore, narratively summarized by study design, covering participants’ characteristics, KD interventions, dropout data, survival, quality of life, metabolic and anthropometric parameters, and safety outcomes [Table 2 and Table 3]. More detailed results are provided in the Supplementary Materials [Table S4].

### 3.2. Methodological Quality of Studies

Of the 23 studies included, two were randomized controlled trials (RCTs) [52,53], eleven were quasi-experimental [17,18,43,44,45,46,47,48,49,50,51], six were case series [37,38,39,40,41,42], and four were case reports [33,34,35,36]. Only four studies included a control group [17,49,52,53].

The methodological quality of the included studies, assessed using the JBI critical appraisal tools, is summarized in Table 4. The quality of the two RCTs [52,53] was rated as low due to inadequate allocation concealment and lack of blinding. Among the eleven quasi-experimental studies, two achieved excellent quality [48,49] and two achieved high quality [17,18]. Most were rated as low-quality studies [43,44,45,47,50,51], mainly due to the absence of control groups and single-time outcome assessment. The study by Martin-McGill et al. 2020 [46] achieved moderate quality for similar limitations.

Among the six case series, three [38,39,40] were rated high/excellent quality, while the remaining three [37,41,42] were classified as low-quality studies because of non-standardized measurements, insufficient statistical analysis, and unclear eligibility criteria.

Of the four case reports, three achieved high-quality scores [33,34,35], whereas one Zuccoli et al., 2010 [36] report was rated as a moderate-quality study due to incomplete reporting of adverse events and take-home messages.

### 3.3. Patient Characteristics

A total number of 306 patients was included throughout the 23 selected studies. The number of participants ranged from 3 to 29 in case series and from 6 to 32 in quasi-experimental studies. The sample size in the RCTs was 50 participants [52,53]. The ages of the patients ranged from 3 to 77 years. Two studies were focused on children aged 3 and 8.5 years old [33] and 4.4, 11.1, and 14.5 years old [41]. Among the nineteen studies that provided exhaustive information regarding participants’ gender, the number of men was 128 (58.7%).

Glioblastomas were the most frequently investigated type of glioma (71.57%), followed by astrocytomas (19.28%), oligodendrogliomas (4.90%), and other gliomas, including intrinsic pontine gliomas (0.98%), gliosarcoma (0.33%), and unspecified low-grade brain tumors (2.94%). Patients with high-grade gliomas (grade III or IV) were present in almost all included studies.

The isocitrate dehydrogenase (IDH) mutation status was specified in eleven studies [34,35,38,39,40,42,44,45,46,50,51] while MGMT promoter methylation status was reported by fourteen studies [18,34,35,36,38,39,40,42,44,46,50,51,52,53].

Three studies [44,46,47] recruited patients with newly diagnosed gliomas, while most participants across the studies had recurrent disease. In the studies by Santos [35,49], all patients had relapsed glioblastomas and had no further standard therapeutic options.

Regarding the treatment of gliomas, all patients in these studies had received conventional therapies (i.e., radiotherapy, chemotherapy) prior to, or during, the dietary interventions.

### 3.4. Dietary Intervention Characteristics

Different Ketogenic Diet (KD) regimens with varying macronutrient compositions were identified across the included studies. Six studies applied the Classic Ketogenic Diet (CKD) as the sole intervention, with ketogenic ratios ranging from 3:1 to 4:1 [17,36,38,42,43,44]. In the case reported by Zuccoli et al. [36], caloric intake was restricted to approximately 600 kcal/day.

One study implemented the Medium-Chain Triglyceride Ketogenic Diet (MCT-KD) [33] and one applied the Modified Atkins Diet (MAD) [18]. Ten studies were classified as using a Modified Ketogenic Diet (MKD) [34,35,37,39,40,45,48,49,52,53].

Several studies employed combined or sequential regimens. Porper et al. [47] used a MAD supplemented with MCTs, while Schreck et al. [50] alternated five days of MAD with two days of restricted CKD (<20% caloric intake, ratio 4:1). Van der Louw et al. [41] implemented a fluid CKD (4:1) for six weeks followed by a solid-food KD with MCT (ratio 1.5–2.0:1); in a pediatric cohort [51], CKD was switched to MCT-KD with a reduced ratio. Martin-McGill et al. [45] compared MCT-KD and MKD in separate patient groups.

Fasting protocols were incorporated alongside MKD in four studies [34,39,52,53].

Sixteen studies specified a predefined intervention duration [18,33,35,38,41,42,43,44,45,46,47,48,49,51,52,53] ranging from 9 days to 12 months.

### 3.5. Patient Dropouts

Of the 306 patients recruited, 62 (20.26%) withdrew from the studies. In two studies, 13 patients died before completion [49,51], but these cases were not classified as dropouts.

The rate of dropout in the studies varied considerably, with the most frequently reported reasons for dropout including dietary restrictions, disease progression, side effects, and concerns regarding quality of life.

The most frequent reason for KD discontinuation was dietary restrictiveness (n = 11), reported across studies employing various ketogenic regimens, including CKD [38,44] MAD [18], MKD [45], MCTKD vs. MKD [46] or MAD + restricted CKD [50]. Tumor progression (n = 10) also contributed to withdrawals in several protocols—CKD [43,44], MKD [45], MCTKD vs. MKD [46], MAD + MCT [47] and MKD + fasting [52]. Adverse effects (n = 9), mainly gastrointestinal symptoms, hyperuricemia, and intolerance, were described in MCT-based or combined protocols [46,47,51]. Reduced quality of life (n = 7) was reported in MKD and mixed MAD + CKD protocols [48,50], while social and personal challenges (n = 5) emerged in CKD + MCT and MKD studies [43,51]. Unspecified reasons represented the largest group (n = 19), mostly in MKD studies [46,49]. Other isolated causes included recruitment into other trials or pre-diet withdrawal [46,52].

### 3.6. Outcomes

#### Survival and Disease Progression

Eleven studies reported data on patient overall survival (OS), testing different dietary interventions [18,34,39,41,44,46,47,48,51,52,53]. The results varied across the studies. In adults, the longest overall survival (OS) was reported by Phillips et al. [34], with 38 months from diagnosis in a 64-year-old woman with IDH-wildtype glioblastoma treated with standard therapy and a three-year intensive dietary program. Klein et al. [44] found a mean OS of 20 months from CKD initiation in newly diagnosed patients and 12.8 months in recurrent cases, corresponding to 21.8 and 25.4 months from diagnosis, respectively. The shortest survival was described by Rieger et al. [48], with a median OS of 32 weeks (range 6–86 weeks) in recurrent glioblastoma treated with MKD.

In pediatric populations, van der Louw et al. [41] reported OS values of 6.4, 16.5, and 18.7 months (mean 13.8) in three children with recurrent pontine glioma treated with CKD + MCT-KD, whereas two astrocytoma cases remained disease-free at four and five years after diagnosis [33].

Progression-free survival (PFS) was reported in eight studies [37,40,44,46,47,48,52,53]. Rieger et al. [48] reported a median PFS of only 5 weeks, while Porper et al. [47] observed a median PFS of 10 months for newly diagnosed disease and 4 months for recurrent disease. Martin-McGill et al. reported a longer mean PFS of 25.8 months [46].

Randomized trials [52,53] found no significant differences in OS or PFS between the MKD-IF and control groups; however, exploratory analysis showed significantly longer survival in patients with glucose levels below the median on day 6 of fasting.

### 3.7. Quality of Life

Only four studies assessed the impact of Ketogenic Diets (KDs) on quality of life (QoL) using standardized questionnaires. Three employed the EORTC QLQ-C30 [46,51,53]; one used the SF-36 survey [40].

In the study by van der Louw et al. [51], QoL remained stable during CKD + MCT-KD treatment, with scores comparable to normative data from the Dutch cancer survivor population. Martin-McGill et al. [46] assessed Global Health Status (GHS) and reported an increase in the MKD group but a decrease in the MCT-KD group after one year; however, participants from both groups described subjective improvements and perceived greater control over their illness. The RCT by Voss et al. [53] found no significant GHS differences between the MKD-IF group and control groups.

In Smith et al. [40], most patients reported perceived improvements in QoL during MKD treatment, although quantitative data were incomplete. Additionally, other studies of MCT-KD [33], CKD [38] and MKD [35] provided qualitative feedback suggesting QoL enhancement during, or after, dietary therapy.

### 3.8. Biochemical Markers

Metabolic parameters related to KDs—blood glucose, ketone levels, and the glucose–ketone index (GKI)—were inconsistently measured and reported across studies.

#### 3.8.1. Blood Glucose

Fourteen studies assessed blood glucose levels. Four trials applying different KD regimens showed non-significant reductions [43,47,48,50], while Voss et al. [52] reported a significant decrease in the MKD-IF group versus controls. In Phillips et al. [39], glucose levels were lowest during fasting and slightly higher during MKD or MKD + fasting phases. Conversely, two studies observed a slight increase [34,38] and one reported no change [33]. Other reports provided mean values only or lacked statistical analysis [35,36,37,44,51].

#### 3.8.2. Ketone Levels

Eighteen of 23 studies reported ketone data. Ten studies measured blood ketones [18,33,34,38,39,40,43,47,51,52], five measured urinary ketones [35,36,45,46,48], one measured both urinary and blood ketones [44]; two studies also included cerebral ketone [17,50] measurements assessed by MRS. Eight studies used ketone levels to monitor adherence [17,18,33,35,45,46,48,50].

Significant increases in blood ketones were observed in two studies [43,47]. Voss et al. [52] again found higher levels in the MKD-IF group versus controls. Klein et al. [44] reported elevated ketone levels in both newly diagnosed and recurrent glioblastoma, with slightly lower values in the latter. Phillips et al. [39] noted peak blood concentrations during fasting (3.52 mmol/L) and the lowest concentrations during MKD (1.11 mmol/L). Two studies evaluated cerebral ketone concentrations: Artzi et al. [17] detected acetoacetate and acetone in both normal and lesional brain tissue after CKD, while Schreck et al. [50] observed increased β-hydroxybutyrate in lesions and acetone bilaterally, indicating altered ketone distribution under MAD + restricted CKD.

#### 3.8.3. Glucose–Ketone Index (GKI)

Only three studies reported GKI data. Phillips et al. [34] observed increasing values over time (mean 3.20, range 1.4–17.2), whereas Panhans et al. [38] noted an initial decline followed by a later rise. In contrast, Phillips et al. [39] found lower GKI values (3.22) during fasting than during KD alone (5.10), reflecting a deeper state of ketosis during fasting.

### 3.9. Anthropometric Outcomes: Weight and BMI

Anthropometric parameters, including body weight and BMI, were reported in 20 studies to evaluate nutritional status and potential unintended effects of Ketogenic Diets (KDs). Five studies assessed body weight only [33,37,42,48,52], 4 studies focused exclusively on BMI [18,38,41,51], and 11 studies evaluated both [34,35,36,39,43,44,45,46,47,49,50].

Overall, 16 studies reported reductions in weight and/or BMI during KD treatment. Statistically significant decreases were observed in four studies applying CKD [43], MKD [48], MAD + restricted CKD [50] and MKD-IF [52]. Four studies reported stable anthropometric measures [42,45,47,49], while no study documented increases in weight or BMI.

### 3.10. Side Effects and Tolerability

Safety and tolerability were assessed across the included studies, with the Ketogenic Diet generally described as well tolerated. Of the 23 studies, 16 explicitly reported adverse effects [18,34,37,38,39,41,42,43,44,45,46,47,48,50,51,52]. Most reported events were mild to moderate in intensity.

Gastrointestinal symptoms were the most frequent adverse effects. Constipation affected 21 patients across ten trials using CKD, MKD, MAD, or mixed regimens [18,37,41,42,44,45,46,47,48,51]. Diarrhea, nausea, and vomiting were also common [33,40,41,43,45,46,47,49,50,51].

Fatigue was reported in six studies, across MKD, CKD, MKD + fasting, CKD + MCT, and MAD + restricted CKD protocols [34,37,39,41,44,50]. Less frequent complaints included loss of appetite [38,47], dizziness [39,44], and headache [50,52]. Metabolic alterations were occasionally observed, including electrolyte imbalances [46], hyperglycemia [43], and elevated cholesterol [47,51].

Seizure recurrence or breakthrough episodes were noted in several trials [39,46,47,50,51,52].

Overall tolerability was reported in 17 of the 23 studies, and 16 described the diet as well tolerated [17,18,33,35,36,37,38,41,44,45,47,48,49,50,52,53]. Only Martin-McGill et al. [46] reported poor tolerability, with participants expressing relief after discontinuation, likely related to the prolonged 12-month intervention.

## 4. Discussion

This systematic review synthesized clinical evidence on the use of Ketogenic Diets (KDs) as adjunctive therapy in patients with glioma. The 23 included studies showed substantial heterogeneity in design, sample size, population characteristics, intervention type, and treatment duration, which hinders direct comparison and data pooling.

Approximately 20% of patients discontinued KD treatment, primarily due to dietary restrictiveness, treatment burden, adverse effects, and non-clinical factors such as social or logistical barriers. Tumor progression was also a frequent reason for withdrawal. These findings highlight the need for individualized KD protocols that incorporate caregiver support, nutritional counseling, and flexible implementation strategies to enhance adherence. Attrition across the studies ultimately reduces sample sizes and introduces selection bias, weakening the overall evidence base and internal validity.

Although preclinical data and mechanistic rationale remain promising, the clinical evidence is generally of low to moderate methodological quality, with a high risk of bias limiting interpretability. Among randomized controlled trials, key weaknesses included the lack of allocation concealment and blinding, increasing the likelihood of performance and detection bias, even if due to the nature of the dietary intervention. Blindness of the participants and personnel blinding was generally not feasible. Unlike pharmacological clinical trials, it is rarely possible to use randomized, double-blind controlled designs in dietary intervention studies, as participants are necessarily aware of the dietary intervention and must be willing and able to adhere to it. Although this may introduce potential selection and adherence biases, it reflects an inherent methodological challenge in studies evaluating dietary interventions. Moreover, most quasi-experimental studies were rated as low quality due to the absence of control groups and repeated outcome assessments, which limits causal inference.

Similarly, many case series lacked robust statistical analysis, standardized outcome measures, and clearly defined inclusion criteria, constraining reproducibility and external validity. While case reports occasionally achieved high reporting quality, their design inherently provides the lowest level of evidence.

### 4.1. Survival and Disease Progression

Among the studies included in this review, different results on OS and PFS were described. These mixed results may be, at least partially, attributed to different characteristics of analyzed populations (such as age, disease stage, tumor-specific features, and concomitant oncology treatment) that may affect patient survival and tumor progression.

In this analysis, the longest survival (thirty-eight months) was observed in a 64-year-old woman who had certain positive prognostic factors, including complete resection and borderline methylation status [34]. Rieger et al. [48] reported the shortest median overall survival (~8 months) among the included studies and suggested that the limited therapeutic effect of MKD might be attributable to the inability to achieve consistent glucose reduction. This was likely influenced by concomitant steroid use and the absence of caloric restriction in their protocol.

Similarly, the RCT evaluating MKD combined with intermittent fasting [52,53] found no significant survival benefit compared with standard diet controls. The lack of improvement was attributed to small sample size, short intervention duration, and differences in prior treatments between groups. Notably, lower blood glucose levels during the fasting phase were associated with more favorable outcomes, underscoring the potential relevance of metabolic control to treatment efficacy [52,53].

However, despite the mixed results, some of the studies included in this review reported an overall survival rate better than the known prognosis of patients with glioma receiving standard therapy alone [54,55,56,57]. This is in line with some previous reviews on this topic [16,58]. Although some case reports and uncontrolled studies described encouraging findings regarding ketogenic dietary interventions in glioma patients, these results should be interpreted with caution, given the methodological weaknesses of these studies, particularly considering that randomized controlled trials reported no significant improvements in survival outcomes [52,53].

A recently published clinical study [59] involving 18 glioblastoma patients reported a three-year survival rate of 66.7% among individuals who adhered to a Mediterranean version of CKD for more than six months. Although not included in our analysis, these findings are noteworthy, particularly when contrasted with outcomes in patients who did not maintain the diet over the same period and with the survival data summarized in this review.

In conclusion, despite these encouraging observations, the small sample sizes, heterogeneity of study populations and dietary protocols, and the absence of control groups in most included studies limit the ability to draw firm conclusions about the effectiveness of Ketogenic Diets in slowing disease progression or improving survival outcomes.

### 4.2. Quality of Life

In this review, only a few studies applied specific tools to evaluate the impact of KDs on the quality of life of patients with glioma. Overall, the results are inconsistent, frequently showing no QoL changes.

Furthermore, some studies reported QoL data without using objective and validated scales; therefore, the results may be subject to interpretation bias.

It is important to note that quality of life is directly influenced by factors other than KD, including symptoms of disease, tumor stage, and adverse events of standard therapy [16].

In conclusion, it is impossible to draw any conclusions about the effectiveness of KDs in enhancing the quality of life of patients with glioma.

### 4.3. Biochemical Markers

Across the various studies, ketone and glucose levels, as well as the Glucose–Ketone index (GKI), varied independently to the specific dietary regimen employed. The effectiveness of the KDs in targeting glioma metabolism appears to be supported by the consistent induction of systemic ketosis in the majority of studies. This is particularly relevant considering the Warburg effect, whereby glioma cells increase glucose consumption and lose the ability to oxidize ketones [60,61,62]. It is noteworthy that the levels of ketones set as the threshold for achieving ketosis varied among the different studies. Moreover, current glioma studies do not establish validated ketone or glucose targets for tumor control. In this systematic review, most studies reported moderate ketone concentrations (around 1.5–3.0 mmol/L). While systemic ketosis was often achieved, brain uptake remains a less evaluated critical issue [17,63,64]. Only two studies directly assessed cerebral ketone presence [17,50]. Across included studies, the majority documented a reduction in blood glucose levels during different KD interventions.

The GKI, which reflects the glucose and ketone balance, has gained attention as a potential biomarker, although its optimal therapeutic threshold in humans remains undefined. Nevertheless, previous studies have suggested that GKI levels approaching 1.0 are potentially therapeutic in managing brain tumor growth when combined with standard therapies [38,65]. Of the three studies that assessed the GKI, only two reported values close to 1 during the dietary intervention, while the third obtained similar values only during the fasting period.

Despite the heterogeneous clinical findings, the biological rationale supporting ketogenic interventions in glioma remains engaging. KD may increase tumor susceptibility to energetic stress by reducing systemic glucose availability while elevating circulating ketone bodies, thereby targeting the metabolic inflexibility associated with the Warburg phenotype in glioma cells [11,13,60,61,62]. In contrast, healthy neural tissue appears more capable of utilizing ketone bodies as an alternative energy substrate, potentially preserving cerebral energy metabolism during treatment [12,13,17]. Moreover, KD leads to increased circulating levels of ketone bodies, including β-hydroxybutyrate (BHB), acetoacetate, and acetone. BHB has been associated with anti-inflammatory effects and protection against oxidative stress, potentially contributing to neuroprotective and metabolic benefits [13]. These mechanisms may theoretically increase tumor susceptibility to energetic stress and enhance responsiveness to standard oncologic therapies, potentially influencing disease progression and life expectancy. However, long-term ketogenic interventions may also influence systemic metabolic pathways. Dietary composition, such as fat quality, may have an impact on lipid metabolism and associated metabolic functions; however, the effects seem to be variable according to the ketogenic dietary formulation and patient characteristics [66,67,68]. Furthermore, intake of soluble fiber and resistant starch may have an impact on gut microbiota composition and related immunometabolic pathways [69]. While Ketogenic Diets have been linked with anti-inflammatory activity and modulation of the gut microbiota, variations in food composition could change microbial diversity and the generation of bioactive metabolites implicated in host metabolic and immune regulation [70,71]. Recent evidence indicates the potential contribution of microbiota-derived metabolites to the biological effects of ketogenic therapies (in preclinical glioma models, as well) [72]. As a result, in interpreting the possible effect of KDs on long-term clinical outcomes and survival, these factors should be taken into account. More studies should be performed on individually tailored supplementation approaches.

### 4.4. Anthropometric Outcomes: Weight and BMI

Previous studies reported that intentional weight loss may, under certain metabolic conditions, exert antitumor pressure via mechanisms such as insulin reduction, glucose limitation, and oxidative stress resistance [62,73].

Across the included studies, ketogenic interventions were consistently associated with modest reductions in, or stabilization of, body weight and BMI, with no evidence of excessive or harmful weight loss. In several trials, these reductions appeared intentional or metabolically favorable, particularly among overweight or obese patients, and were accompanied by decreases in visceral adiposity and improved metabolic profiles [36,39,43]. For instance, Phillips et al. [39] reported an average weight loss of 8.4 ± 6.9 kg (11.2%) and a BMI decrease of 2.9 ± 2.3 kg/m^2^ over 161 days, classified as a common terminology criteria of adverse events (CTCAE) grade 2 event, but resulting in BMI normalization among overweight participants. Nonetheless, unintended weight loss remains a critical concern in oncology, as it may contribute to malnutrition, sarcopenia, and cancer-related cachexia [74]. Other studies, including those by Martin-McGill et al. [45] and Porper et al. [47], observed minimal and statistically nonsignificant anthropometric changes, reinforcing the safety profile of well-managed KDs. However, it is important to emphasize that, in patients with GBM, assessing unintentional weight loss based solely on static parameters, such as weight or BMI, can be highly misleading. Conventional corticosteroid treatments can mask systemic muscle atrophy by altering body composition and promoting fluid retention. However, underlying sarcopenia and inflammatory cachexia are clinically significant, since they act as strong risk factors for early treatment discontinuation and symptoms of cerebral oedema. Furthermore, patients in the lowest quartiles of muscle mass have significantly shorter progression-free survival, regardless of standard tumor characteristics. This highlights the need to monitor and intervene in order to prevent muscle loss alongside the use of traditional anthropometric measures to improve therapeutic management [75,76,77,78].

### 4.5. Side Effect and Tolerability

The literature has documented a wider and transient spectrum of the adverse effects of KD [21]. Our results indicate that KD interventions are generally safe and well tolerated in patients with brain tumors. Among the studies reviewed, 16 (69.6%) reported side effects that were predominantly mild to moderate. Constipation was the most frequent symptom, followed by fatigue, diarrhea, nausea and vomiting. Seizures were reported in six studies, but in at least one case were attributed to tumor progression rather than the diet [39].

Specific contraindications to KDs exist, particularly in metabolic or hepatic disorders [79], underscoring the need for individualized assessment and monitoring.

The studies included in this review showed that KDs were well tolerated by most individuals, even those on oncology treatment. Only one study described poor tolerability, mainly due to the prolonged duration of the intervention [46].

These findings align with prior evidence suggesting that KDs, when properly managed, do not interfere with standard oncologic care [16,58].

### 4.6. Study Limitations

This review presents several limitations. The substantial heterogeneity in patient characteristics (e.g., age, sex, tumor type and stage, prior or ongoing therapy, surgical history) and in dietary interventions (type, duration, and monitoring methods) limited the ability to synthesize findings and precluded meta-analysis. The included studies assessed a wide range of KD-related outcomes using inconsistent methodologies, further reducing comparability. Small sample sizes, absence of control groups, and non-randomized designs frequently compromised methodological quality and strength of the evidence. Additionally, grey literature was not included in the search strategy, which may have led to the omission of relevant studies. Finally, given the substantial methodological and clinical heterogeneity across included studies and the predominance of non-comparative designs, a formal certainty-of-evidence assessment using GRADE was not performed. Collectively, these factors underscore the need for well-designed, adequately powered randomized controlled trials employing standardized KD protocols and uniform outcome measures in homogeneous glioma populations. Future research should prioritize adequately powered randomized controlled trials, standardized dietary protocols and uniform outcome measures to better clarify the role of KD as an adjunctive intervention in malignant glioma. Furthermore, concomitant pharmacological treatments—particularly corticosteroid therapy—represent a significant confounding variable in the assessment of Ketogenic Diet (KD) efficacy, given their well-documented effects on glycemic regulation and immune function.

Another important limitation of these studies is that the attribution of adverse events remains unclear, as they may be related to the complexity of the underlying tumor, concomitant treatments or medications, rather than to the Ketogenic Diet itself. Future studies should incorporate standardized adverse event attribution methods (e.g., classification of events as unrelated, possible, or probable) and evaluation of temporal relationships between Ketogenic Diet exposure and adverse event occurrence or resolution, in order to better disentangle diet-related toxicities from symptoms related to the tumor itself or concomitant therapies.

## 5. Conclusions

Current evidence suggests that ketogenic dietary interventions in patients with glioma are generally feasible and may be safely implemented under appropriate clinical and nutritional supervision. Most studies reported acceptable tolerability and predominantly mild adverse events; however, adherence remains a major challenge, especially in long-term or highly restrictive protocols. Therefore, ketogenic dietary interventions should be implemented within a personalized and multidisciplinary framework, taking into account tumor histology and molecular profile, concomitant oncological treatments, patient nutritional and metabolic status, risk of sarcopenia, clinical condition, and diet adherence. Many questions regarding the optimal dietary protocol, treatment duration, long-term safety, effects on patient nutritional status, and potential impact on patient quality of life and survival outcomes remain open. Given the methodological challenges inherent to nutrition research, the clinical complexity of gliomas, and the promising potential of ketogenic dietary interventions, further well-designed studies are needed to clarify their clinical utility in glioma treatment.

## Figures and Tables

**Table 1:** PICOS eligibility criteria.

Parameter	Inclusion Criteria	Exclusion Criteria
Population	Patients with malignant glioma	Patients with different tumors
Intervention	Any type of Ketogenic Diets (applied before, during or after standard therapies)	Other dietary regimens
Comparator	Patients treated with different type of diet	
Outcome	Dropout; survival, quality of life, biochemical markers, anthropometric measures, safety, tolerability	N/A
Study design	All study designs (e.g., Randomized Controlled Trials, Quasi-Experimental, Clinical Trial, Case–Control, Case Series, Case Report)	Systematic review, meta-analysis, letters to editors, guidelines, book chapters, conference abstracts, gray literature.

N/A not available.

**Table 2:** Description of studies and interventions in glioma patients.

Author, Year, Country, Study Design	Population (N, Age, Gender)	Cancer Type	IDH Mutation Status	MGMT Promoter Methylation	Dietary Intervention (KD Type and Duration)	N. Dropout and Main Reasons
Nebeling et al. 1995, USA, Case Report [33]	N = 2 3 yr; 8.5 yr F	Anaplastic astrocytoma grade IV cerebellar astrocytoma grade III	NA	NA	MCT KD Duration: 8 wk	
Phillips et al. 2024, New Zealand, Case Report [34]	N = 1 64 yr F	GBM	wt	Met	MKD + 7-day fluid-only fasts Duration: 3 yr	
Santos et al. 2017, Brazil, Case Report [35]	N = 1 54 yr M	GBM	wt	unMet	MKD Duration: 3 mo	
Zuccoli et al. 2010, Italy, Case Report [36]	N = 1 64 yr F	GBM	NA	Met	restricted CKD 4:1 (about 600 kcal/day) Duration: 2 mo	
Champ et al. 2014, USA, Retrospective Case Series [37]	N = 6 Mean age (range): 53.8 (34–62) yr Gender: NA	GBM grade III–IV	NA	NA	MKD Duration: 3–12 mo	None
Panhans et al. 2020, USA, Retrospective Case Series [38]	N = 12 Median age (range): 43.5 (27–60) yr 10 M (83%)	6 GBM grade IV 5 astrocytoma grade II–III 1 oligodendroglioma grade II	6 wt 6 mut	6 Met 4 unMet 2 NA	CKD 3:1 Duration: 4 mo	2/12 Difficulties during social events Diet perceived as “too restrictive”
Phillips et al. 2022, New Zealand, Prospective Case Series [39]	N = 10 Mean age (SD): 58.0 (11.9) yr 7 M (70%)	GBM	9 wt 1 NA	5 Met 3 unMet 2 NA	MKD + 5–7 fasting day (every 1–2 mo) Duration: 5–6 mo	None
Smith et al. 2022, USA, Retrospective Case Series [40]	N = 16 Mean age (range): 48.3 (25–75) yr 10 M (62.5%)	4 GBM grade IV 7 oligodendroglioma (grade II–III–IV) 1 gliosarcoma grade IV 4 astrocytoma grade II	12 wt 3 mut 1 NA	8 Met 3 unMet 5 NA	MKD Duration: 3–48 mo	NA
van der Louw et al. 2018, Netherlands, Prospective Case Series [41]	N = 3 Mean age (SD): 9.9 (5) yr 3 M (100%)	Diffuse intrinsic pontine glioma (DIPG) recurrent	NA	NA	CKD 4:1 + MCT KD (1.5:1–2.0:1) Duration: 3 mo	None
Zapata Laguado et al. 2024, Colombia, Prospective Case Series [42]	N = 29 Mean age (range): 45 (17–64) yr 13 M (44.8%)	15 GBM 6 astrocytoma grade III 5 oligodendroglioma grade III 3 pilocytic astrocytoma grade III	3 wt 18 mut 8 NA	3 Met 19 unMet 7 NA	CKD (range 1.4:1–3:1) Duration: 12 mo (follow-up)	NA
Artzi et al. 2017, Israel, Quasi-Experimental [17]	N = 9 CKD group = 5 Mean age (range): 51 (37–69) yr 2 M (40%) Control group = 4 Mean age (range): 46 (27–64) yr 3 M (75%)	CKD group: 4 GBM 1 gliomatosis cerebri (low-grade astrocytoma) Control group: 4 GBM	NA	NA	CKD 4:1 Duration: 2–31+ mo	NA
Foppiani et al. 2021, Italy, Quasi-Experimental [43]	N = 12 Mean age (range): 41.4 (22–54) yr 6 M (50%)	11 GBM grade V 1 anaplastic oligodendroglioma III	NA	NA	CKD 3:1 Duration: 1 mo	5/12 Rapid tumor progression Discontinuation after 1 wk
Klein et al. 2020, USA, Quasi-Experimental [44]	N = 8 Mean age (range): 49.8 (40–64) yr 6 M (75%)	Group 1: 4 newly diagnosed GBM Group 2: 4 progressive or recurrent GBM	6 wt 2 NA	2 Met 5 unMet 1 NA	CKD 4:1 or 3:1 Duration: 6 mo	3/8 (group 2) Tumor progression Dietary restrictiveness
Martin-McGill et al. 2018, UK, Quasi-Experimental [45]	N = 6 Mean age (range): 47.5 (34–66) yr 6 M (100%)	5 GBM grade IV 1 anaplastic astrocytoma grade III	5 wt 1 mut	NA	MKD Duration: 3 mo	2/6 Clinical deterioration Dietary preferences
Martin-McGill et al. 2020, UK, Quasi-Experimental [46]	N = 12 MCTKD group = 6 Mean age: 53 yr 5 M (83.3%) MKD group = 6 Mean age: 58.5 yr 3 M (50%)	GBM grade IV new diagnosis	MCTKD group: 5 wt 1 mut MKD group: 5 wt 1 mut	MCTKD group: 1 Met 5 unMet MKD group: 3 Met 2 unMet 1 NA	MCTKD vs MKD Duration: 12 mo	9/12 Withdrawal of consent Dietary burden Recruitment to another trial Tumor progression Nausea Gastrointestinal side effects
Porper et al. 2021, Israel, Quasi-Experimental [47]	N = 13 Mean age (range): 61 (52–74) yr 8 M (62%)	5 GBM + 2 anaplastic astrocytoma. Recurrent 5 GBM + 1 anaplastic astrocytoma New Diagnosis (grade III–IV)	NA	NA	MAD + MCT Duration: 8 wk	5/13 Hyperuricemia Seizures Disease progression Anorexia, nausea, and difficulty meeting dietary goals
Rieger et al. 2014, Germany, Quasi-Experimental [48]	N = 20 Mean age (range): 57 (30–72) yr 7 M (35%)	GBM recurrent IV grade	NA	NA	MKD (CHO < 60 g) Duration: 16 wk	3/20 Reduced quality of life due to CHO restriction
Santos et al. 2018, Brazil, Quasi-Experimental [49]	N = 32 MKD group = 17 Median age (range): 53 (31–61) yr Control group = 15 Median age (range): 48 (27–55) yr Gender = NA	GBM recurrent	NA	NA	MKD Duration: 3 mo	3/17 (MKD group) Non-adherence to the diet
Schreck et al. 2021, USA, Quasi-Experimental [50]	N = 25 Mean age (SD): 50.1 (12.7) yr 13 M (52%)	Astrocytoma grade II–III–IV	11 wt 12 mut 2 NA	11 Met 7 unMet 7 NA	MAD 5 days + restricted 4:1 CKD (Ketocal) 2 days Duration: 8 wk	4/25 Fatigue Difficulty planning meals Weight loss Decreased quality of life
van der Louw et al. 2019, Netherlands, Quasi-Experimental [51]	N = 9 Median age (IQR): 53.8 (22.5) yr 8 M (89%)	GBM	9 wt	2 unMet 7 NA	CKD 4:1 + MCTKD Duration: 14 wk	3/9 Family issues Intolerance to MCT emulsion Non-compliance
Woodhouse et al. 2019, USA, Quasi-Experimental [18]	N = 29 Median Age (range): 52.9 (30.8–76.7) yr 17 M (57%)	1 oligodendroglioma grade II 9 astrocytoma grade II–III 19 GBM	NA	13 Met 12 unMet 4 NA	MAD Duration: 6 wk	1/29 Diet perceived as too restrictive
Voss et al. 2020, Germany, RCT [52]	N = 50 MKD-IF group = 25 Median age (range): 56 (39–71) yr Control group = 25 Median age (range): 58 (26–75) yr Gender: NA	MKD-IF group: 23 GMB+ 2 Low-grade tumors Control group: 18 GBM + 7 Low-grade tumors	NA	MKD-IF group: 11 Met 11 unMet 3 NA Control group: 11 Met 10 unMet 4 NA	MKD-IF group: 3 days MKD + 3 days fasting + 3 days MKD Control group: standard diet Duration: 9 days	8/50 (5 MKD-IF group, 3 Control group) Withdrawal of consent Clinical deterioration
Voss et al. 2022, Germany, RCT [53]	N = 50 MKD-IF group = 25 Median age (range): 56 (39–71) yr Control group = 25 Median age (range): 58 (26–75) yr Gender: NA	MKD-IF group: 23 GMB + 2 Low-grade tumors Control group: 18 GBM + 7 Low-grade tumors	NA	MKD-IF group: 11 Met 11 unMet 3 NA Control group: 11 Met 10 unMet 4 NA	MKD-IF group: 3 days MKD + 3 days fasting + 3 days MKD. Control group: standard diet Duration: 9 days	30/50 Only 20 pt completed the trial

Legend: CHO Carbohydrates; CKD classic ketogenic diet; F female; IDH isocitrate dehydrogenase; IF intermittent fasting; M male; MAD modified Atkins diet; MCT KD medium-chain triglyceride ketogenic diet; MKD modified ketogenic diet; Met methylated; MGMT O6-methylguanine DNA methyltransferase; MO months; MUT mutant; NA not available; unMet unmethylated; WK weeks; WT wildtype; YR years.

**Table 3:** Summary of study results on survival, quality of life, biochemical markers, anthropometric measures, safety and tolerability.

Author, Year, Country, Study Design	Main Results				
	Survival and Disease Progression	Quality of Life	Biochemical Markers	Weight and BMI	Safety and Tolerability of KDs
Nebeling et al. 1995, USA, Case Report [33]	NA	Pt 1 Overall improvement	Blood glucose stable/slightly reduced Blood ketones increased	Weight stabilized	Side Effect: NA Tolerability: well tolerated
Phillips et al. 2024, New Zealand, Case Report [34]	OS from diagnosis: 38 mo	NA	Blood glucose stable/slightly increased Blood ketone stable/slightly reduced GKI increased	Weight reduced during the first yr, partial recovery over time BMI reduced during the first yr, partial recovery over time	Side Effect: mild fatigue, diarrhea, cold intolerance were linked to prolonged fasts (No diet-related) Tolerability: NA
Santos et al. 2017, Brazil, Case Report [35]	NA	Overall improvement	Fasting glucose slightly reduced Urine ketones stable during the treatment	Weight slightly reduced BMI slightly reduced	Side Effect: NA Tolerability: well tolerated
Zuccoli et al. 2010, Italy, Case Report [36]	NA	NA	Blood glucose maintained at low/stable levels during diet Urine ketones achieved	Weight marked reduced BMI marked reduced	Side Effect: NA Tolerability: well tolerated
Champ et al. 2014, USA, Retrospective Case Series [37]	PFS mean (range): 10.3 (9.4–17) mo	NA	Blood glucose variable during the study	Weight reduced	Side Effect: CTCAE grade I-II fatigue (5 pt); constipation (1 pt), weight loss (1 pt), nephrolithiasis (1 pt), alopecia (6 pt); DVT (1 pt) Tolerability: well tolerated
Panhans et al. 2020, USA, Retrospective Case Series [38]	NA	improvement in energy, mood, neuro-cognitive function, and overall wellbeing and symptoms	Blood glucose stable Blood ketone transient increase at 30 days GKI reduced	BMI reduced	Side Effect: increasing severity of headaches (2 pt), appetite (4 pt) Tolerability: well tolerated
Phillips et al. 2022, New Zealand, Prospective Case Series [39]	Survival median: 13 mo	NA	Blood glucose lower values during fast Blood ketones higher values during fast GKI lower values during fast	Weight reduced BMI reduced	Side Effect: CTCAE grade I-II fatigue, irritability, lightheadedness, 4 pt seizures due to tumor progression Tolerability: NA
Smith et al., 2022, USA, Retrospective Case Series [40]	PFS mean (SD): 20.0 (14.4) mo	36-item Short Form Survey Improvement	Blood BHB achieved	NA	NA
van der Louw et al. 2018, Netherlands, Prospective Case Series [41]	OS from diagnosis mean (range): 13.8 (6.4–18.7) mo	NA	NA	SD score for weight for height mixed changes among pt	Side Effect: CTCAE fatigue, vomiting, food refusal, constipation, inability to swallow Tolerability: well tolerated. Ketogenic snacks improved the tolerance
Zapata Laguado et al. 2024, Colombia, Prospective Case Series [42]	NA	NA	NA	Weight maintained (24 pt), reduced (5 pt)	Side Effect: gastrointestinal symptoms grade I diarrhea (5 pt), vomiting (3 pt), constipation (2 pt) Tolerability: NA
Artzi et al. 2017, Israel, Quasi-Experimental [17]	NA	NA	Urine ketone achieved after 1 mo Cerebral ketones metabolism on MRS AcAc and/or acetone were only detected on MRS in 1 pt from the CKD group	NA	Side Effect: NA Tolerability: well tolerated in 4/5 pt
Foppiani et al. 2021, Italy, Quasi-Experimental [43]	NA	NA	Blood pre-prandial glycemia stable/slightly reduced *p* = 0.643 Blood Pre-prandial Ketonemia increased *p* = 0.024 *	Weight reduced *p* = 0.013 * BMI reduced *p* = 0.013 *	Side Effect: frequent mild hyperglycemia events (max value 115 mg/dL) Tolerability: NA
Klein et al. 2020, USA, Quasi-Experimental [44]	OS from diagnosis mean (range) longer in Group 2: 25.4 (13.9–38.7) mo OS after start of the diet mean (range) longer in Group 1: 20 (9.5–27) mo PFS mean (range) longer in Group 2: 3.9 (0.6–9.1) mo	NA	FPG during diet higher in Group 2 Blood BHB during diet higher in Group 1 Urine ketone during diet higher in Group 1	Weight reduced BMI reduced	Side Effect: TEAEs mild-transient weight loss and hunger (6 pt), nausea (3 pt), dizziness (2 pt), fatigue (1 pt), constipation (1 pt) Tolerability: well tolerated
Martin-McGill et al. 2018, UK, Quasi-Experimental [45]	NA	NA	Urine ketone achieved within 1 wk. 3 pt maintained ketosis during the study	Weight stable *p* = 0.71 BMI stable *p* = 0.75	Side Effect: constipation (2 pt) Tolerability: well tolerated
Martin-McGill et al. 2020, UK, Quasi-Experimental [46]	OS mean (range) from surgery longer in MCT KD: 59.3 (35.4–83.6) wk PFS mean (range) longer in MCT KD: 25.82 (14–44.4) wk	EORTC QLQ-C30 (GHS) GHS improved in the MKD (1 pt) and worsened in the MCT KD (2 pt) Qualitative interviews both groups reported to experiencing a ‘fantastic quality of life’	Urine ketone achieved in both groups during the first 6 wk and maintained up to 50 wk	Weight reduced in both groups BMI reduced in both groups	Side Effect: MCT KD group: CTCAE grade I hypokalemia (2 pt), hypernatremia (1 pt), hypocalcemia (1 pt), partial seizure (1 pt), diarrhea (1 pt), nausea (1 pt), vomiting (1 pt), dyspepsia (2 pt), constipation (1 pt) MKD group: CTCAE grade I vomiting (1 pt), dry mouth (1 pt), constipation (1 pt) Tolerability: Not well tolerated
Porper et al. 2021, Israel, Quasi-Experimental [47]	OS median longer in New diagnosis: 21 mo PFS median longer in New diagnosis: 10 mo	NA	Blood glucose reduced *p* = 0.4 Blood BHB increased *p* = 0.006 *	Weight stable *p* = 0.4 BMI stable *p* = 0.3	Side Effect: CTCAE grade I–III in 2 pt nausea and asymptomatic hyperuricemia, anorexia (6 pt), nausea (5 pt), weight loss (1 pt), vomiting (3 pt), constipation (3 pt), diarrhea (1 pt), hiccups (1 pt), hypercholesterolemia (8 pt), seizures (1 pt) Tolerability well tolerated
Rieger et al. 2014, Germany, Quasi-Experimental [48]	OS median (range) after start of the diet: 32 (6–86+) wk PFS median (range) 5 (3–13) wk	NA	Blood glucose reduced Urine Ketone almost all pt maintained ketosis during the study	Weight reduced statistically significant weight loss of ~2.2% during the diet	Side Effect: not attributable to the diet. Few pt reported diarrhea, constipation, hunger, demand for glucose Tolerability: well tolerated
Santos et al. 2018, Brazil, Quasi-Experimental [49]	NA	NA	NA	Weight stable BMI stable	Side Effect: NA Tolerability: well tolerated
Schreck et al. 2021, USA, Quasi-Experimental [50]	NA	NA	Blood fasting glucose slightly reduced *p* = 0.057 Urine ketone 80% pt maintained ketosis Cerebral ketones metabolism on MRS increases in BHB and acetone brain concentrations in both lesional (bHB *p* = 0.011, acetone *p* = 0.012) and contralateral (BHB *p* = 0.031, acetone *p* = 0.005. Average ketonuria correlated with cerebral ketones in lesional and contralateral brain (BHB *p* = 0.05)	Weight reduced *p* =< 0.0001 * BMI reduced *p* =< 0.0001 *	Side Effect: CTCAE grade I–III leukopenia (3 pt), nausea (2 pt), colitis (1 pt), diarrhea (1 pt), fatigue (1 pt), headache (1 pt), myalgias (1 pt), leukocytosis (1 pt), seizure (1 pt), neutropenia (1 pt possibly related) Tolerability: well tolerated
van der Louw et al. 2019, Netherlands, Quasi-Experimental [51]	OS median (IQR) from diagnosis: 12.8 (12.3–17.7) mo	EORTC QLQ-C-30 outcomes did not change essentially during the study	Blood glucose higher in MCT KD Blood ketone 9 pt reached adequate ketosis higher in CKD	BMI slightly reduced	Side Effect: CTCAE Grade I–II Constipation (7 pt), nausea/vomiting (2 pt), hypercholesterolemia (1 pt), hypoglycemia (1 pt), low carnitine (1 pt), diarrhea (1 pt), hallucinations (1 pt), allergic reaction (1 pt), wound infection (1 pt) Severe AEs: Seizures (3 pt), ambulatory issues (1 pt), recurrent wound infection (1 pt). Tolerability: NA
Woodhouse et al. 2019, USA, Quasi-Experimental [18]	2-year OS: 4 of 15 (26.7%)	NA	Blood BHB were achieved in 23 pt	BMI reduced 25 pt (1 underweight pt); increase 3 pt; No change 1 pt	Side Effect: Grade II constipation (1 pt) Tolerability: well tolerated
Voss et al. 2020, Germany, RCT [52]	OS median (95% CI): No significant difference between MKD-IF and Control group (*p* = 0.978) PFS median (95% IC) No significant difference between MKD-IF and Control group (*p* = 0.729)	NA	Blood glucose significant reduction between groups, higher reduction in the MKD-IF group (*p* < 0.01) * Ketone significant increase between groups. Higher increase in the MKD-IF group while no change in the Controls (*p* < 0.01) *	Weight significant reduction between groups, higher reduction in the MKD-IF group (*p* = 0.008) *	Side Effect: headache, nausea, seizure possible epileptic seizures with short-lasting aphasia in MKD-IF (4 pt); Control group (5 pt) Tolerability: well tolerated
Voss et al. 2022, Germany, RCT [53]	OS mean (95% CI): No significant difference between MKD-IF and Control group (*p* = 0.965) PFS mean: No significant difference between MKD-IF and Control group (*p* = 0.845). Longer PFS was observed in MKD-IF patients with lower glucose levels on day 6 (*p* = 0.014)	EORTC QLQ-C30 (GHS) no significant difference among MKD-IF group and control group during treatment or at later follow-up 1 mo later	NA	NA	Side Effect: NA Tolerability: well tolerated

Legend: AcAc acetoacetate; AEs adverse effects; BHB beta-hydroxy butyrate; BMI body mass index; CKD classic ketogenic diet; CTCAE common terminology criteria of adverse events; DVT deep venous thrombosis; EORTC-QLQ-C30 European Organization for Research and Treatment of Cancer Quality of Life Questionnaire Core 30; FPG fasting plasma glucose; GHS Global Health Status; GKI glucose–ketone index; IQR interquartile range; IF intermittent fasting; MAD modified Atkins diet; MCT KD medium-chain triglyceride ketogenic diet; MKD modified ketogenic diet; MO months; MRS Magnetic resonance spectroscopy; OS overall survival; NA not available; pt patient; PFS progression free survival; SD standard deviation; SE side effects; TEAE treatment emergent adverse event; wk weeks. * represent statistically significant results with *p* < 0.05.

**Table 4:** Quality score of JBI critical appraisal checklist.

Authors	Study Design	JBI Quality Score
Nebeling et al. 1995 [33]	Case report	100%
Phillips et al. 2024 [34]	Case report	100%
Santos et al. 2017 [35]	Case report	100%
Zuccoli et al. 2010 [36]	Case report	75%
Phillips et al. 2022 [39]	Case series	100%
Panhans et al. 2020 [38]	Case series	90%
Smith et al.2022 [40]	Case series	80%
van der Louw et al. 2018 [41]	Case series	66.6%
Champ et al. 2014 [37]	Case series	60%
Zapata Laguado et al. 2024 [42]	Case series	50%
Rieger et al. 2014 [48]	Quasi-experimental	100%
Santos et al. 2018 [49]	Quasi-experimental	100%
Artzi et al. 2017 [17]	Quasi-experimental	88.8%
Woodhouse et al. 2019 [18]	Quasi-experimental	83.3%
Foppiani et al. 2021 [43]	Quasi-experimental	66.6%
Martin-McGill et al. 2020 [46]	Quasi-experimental	77.7%
Klein et al. 2020 [44]	Quasi-experimental	66.6%
Martin-McGill et al. 2018 [45]	Quasi-experimental	66.6%
Schreck et al. 2021 [50]	Quasi-experimental	66.6%
Porper et al. 2021 [47]	Quasi-experimental	55.5%
van der Louw et al. 2019 [51]	Quasi-experimental	55.5%
Voss et al. 2022 [53]	RCT	61.5%
Voss et al. 2020 [52]	RCT	53.8%

JBI: Joanna Briggs Institute, <70% low quality, 70–79% medium–high quality, 80–90% high quality, >90% excellent quality.

## Data Availability

The original contributions presented in the study are included in the article/Supplementary Material, further inquiries can be directed to the corresponding author.

## Supplementary Materials

The following supporting information can be downloaded at: https://www.mdpi.com/article/10.3390/nu18132166/s1, Table S1. Electronic search strategy; Table S2. KDs implemented in the included studies; Table S3. Studies excluded with reason after reading the full text; Table S4. Overview of study results on survival, quality of life, biochemical markers, anthropometric measures, safety and tolerability.
