# Supplementary material for: Ketogenic Diet in the Treatment of Malignant Gliomas: A Systematic Review
Source: Nutrients. 2026 Jul 3;18(13):2166. doi: 10.3390/nu18132166 (PMC13363701; doi:10.3390/nu18132166)
Supplement: Supplementary file 1 [file nutrients-18-02166-s001.zip › nutrients-4318061-supplementary.pdf]

Table S1 Electronic search strategy

Database	Search strategy
MEDLINE (PubMed)	(("glioma" [MeSH Terms])) AND (("Diet, Ketogenic" [MeSH Terms]) OR ("keto-diet*") OR (ketogen*) OR ("Keto*") OR ("low carb*") OR ("high-fat"))
Embase	((glioma/exp)) AND (('Diet, Ketogenic'/exp) OR (keto-diet*) OR (ketogen*) OR (Keto*) OR ('low carb*') OR (high-fat))
Cochrane Library	(("glioma")) AND (("Diet, Ketogenic") OR (keto-diet*) OR (ketogen*) OR ("Keto*") OR (low carb*) OR ("high-fat"))

Table S2 KDs implemented in the included studies

Term	Abbreviation	Composition
Classic Ketogenic Diet	CKD	Up to 90% fat, 4% carbohydrate, 7% protein
Medium-Chain Triglycerides Ketogenic Diet	MCT KD	70% fat (of which 30-40% MCT) 20% carbohydrates, 10% protein
Modified Atkins Diet	MAD	65-70% fat, 5% carbohydrate, 25% protein
Modified Ketogenic Diet	MKD	70-75% fat, 5% carbohydrate, protein ad libitum

Table S3. Studies excluded with reason after reading the full text

Author/Year	Title	Reason for exclusion
Ebrahimpour-Koujan (2019)	Adherence to low carbohydrate diet in relation to glioma: A case-control study	Ineligible Outcome
Nikolai (2013)	Energy restricted ketogenic diet (ERKD) treatment for advanced glioblastoma multiforme (GBM): Case report	Full text not available in any format
Perez (2021)	Ketogenic diet treatment in diffuse intrinsic pontine glioma in children: Retrospective analysis of feasibility, safety, and survival data	Review based on retrospective studies
Schwartz (2015)	Treatment of glioma patients with ketogenic diets: Report of two cases treated with an IRB-approved energy-restricted ketogenic diet protocol and review of the literature	Literature review
Santos (2020)	Adjuvant effect of low-carbohydrate diet on outcomes of patients with recurrent glioblastoma under intranasal perillyl alcohol therapy	Different type of diet
Tamas (2023)	The Role of Ketone Bodies in Treatment Individualization of Glioblastoma Patients	Different type of diet

Table S4 Overview of study results on survival, quality of life, biochemical markers, anthropometric measures, safety and tolerability

Author, Year, Country, Study design [REF]	Main Results				
	**Survival and disease progression**	**Quality of life**	**Biochemical markers**	**Weight and BMI**	**Safety and tolerability of KDs**
Nebeling et al. 1995, USA, Case Report [32]	NA	Pt 1<br>Overall improvement	**Blood glucose** (mmol/L) mean<br>Pt 1 Baseline 5.5; End of the study 5<br>Pt 2 Baseline 4.5; End of the study 4<br>**Blood ketones** (mmol/L) mean<br>Pt 1 Baseline 0; End of the study 4.8<br>Pt 2 Baseline 0; End of the study 5	**Body weights** were stabilized thereby reversing the weight loss experienced by both patients prior to the start of the study<br>Pt 1 Kg 11 ± 0.5; Pt 2 Kg 24 ± 0.3	**Side Effect**<br>NA<br>**Tolerability**<br>Well tolerated
Phillips et al. 2024, New Zealand, Case Report [33]	**OS** from diagnosis<br>38 mo	NA	**Blood glucose** (mmol/L) mean (SD)<br>I yr 4.65 (0.38); II yr 4.68 (0.19); III yr 5.24 (0.62)<br>**Blood ketones** (mmol/L) mean (SD)<br>I yr 2.82 (1.43); II yr 2.32 (0.67); III yr 1.64 (0.65)<br>**GKI** mean (range)<br>I yr 1.65 (0.52 - 5.97); II yr 2.02 (1.16 -5.38); III yr 3.20 (1.14-17.20)	**Weight** (Kg)<br>Baseline77.5; I yr: 54.64; III yr: 68<br>**BMI** (kg/m2)<br>Baseline; 28,1; I yr: 20.5; III yr: 24.7	**Side Effect**<br>Mild fatigue, diarrhea, cold intolerance were linked to prolonged fasts (No diet-related)<br>**Tolerability**<br>NA
Santos et al. 2017, Brazil, Case Report [34]	NA	Overall improvement	**Fasting glucose** (mg/dL) mean<br>Baseline 77; End of the study 74<br>**Urine ketones** (mean) mg/dL<br>5–15 mg/dL during the treatment	**Weight** (Kg)<br>Baseline 68.7; End of the study 65.7<br>**BMI** (kg/m2)<br>Baseline 23.7; End of the study 22.7	**Side Effect**<br>No SE reported<br>**Tolerability**<br>The combination treatment was well tolerated
Zuccoli et al. 2010, Italy, Case Report [35]	NA	NA	**Blood glucose** (mmol/L)<br>during diet 4-3.5<br>**Urine ketones** (mmol/L)<br>during diet 2.5	**Weight** (Kg)<br>Baseline 64; After 14 days R-KD 55<br>**BMI** (kg/m2)<br>Baseline 25.6; After 14 days R-KD 22	**Side Effect**<br>No SE reported<br>**Tolerability**<br>Well tolerated

Champ et al. 2014, USA, Retrospective study Case Series [36]	**PFS** mean (range)<br>10.3 (9.4-17) mo	NA	**Blood Glucose** (mg/dL) mean (range)<br>122 (83–278)	**Weight** (Kg) mean<br>Baseline 85.7; End of the study 77.7	**Side Effect**<br>CTCAE grade I fatigue (4 pt); constipation (1 pt), weight loss (1 pt), nephrolithiasis (1 pt)<br>CTCAE grade II fatigue (1 pt); Alopecia (6 pt); DVT (1 pt)<br>**Tolerability**<br>Well tolerated
Panhans et al. 2020, USA, Retrospective Case Series [37]	NA	Notable improvement in energy, mood, neuro-cognitive function, overall well-being and symptoms	**Blood glucose** (mmol/dL) mean (range)<br>Baseline 4.7 (3.7-5.5); At 30 days 4.6 (3.9-5.5); End of the study 4.8 (3.8-5.8);<br>**Blood Ketones** (mmol/dL) mean (range)<br>Baseline 1.7 (0.2-5.5); At 30 days 3 (1.7-4.4); End of the study 1.7 (0.9-3)<br>**GKI**<br>Baseline 9.3 (0.57-28.8); At 30 days 1.7 (0.93-2.9); End of the study 3.4 (1.7-6)	**BMI** (kg/m2) mean (range)<br>Baseline 25.5 (21.3-31.6); At 30 days 25.3 (21.1-28.5); At the end 23.7 (18.7-30.1)	**Side Effect**<br>Increasing severity of headaches (2 pt), appetite (4 pt)<br>**Tolerability**<br>Well tolerated
Phillips et al. 2022, New Zealand, Prospective Case Series [38]	**Survival** median<br>13 mo	NA	**Blood glucose** (mmol/L) mean (SD)<br>Fasts+ MKD 5.44 (0.55); Fast 4.50 (0.68); MKD 5.66 (0.46)<br>**Blood ketones** (mmol/L) mean (SD)<br>Fasts+ MKD 1.69 (0.62); Fast 3.52 (0.99); MKD 1.11 (0.52)<br>**GKI** (mean)<br>Fasts+ MKD 3.22; Fast 1.28; MKD 5.10	**Weight** (Kg) mean (SD)<br>Baseline 75.1 (13.8); End of the study 66.7 (14.0)<br>**BMI** (kg/m2) mean (SD)<br>Baseline of 25.4 (4.3); End of the study 22.5 (4.2)	**Side Effect**<br>CTCAE grade I-II fatigue, irritability, lightheadedness, 4 pt seizures due to tumor progression<br>**Tolerability**<br>NA
Smith et al, 2022, USA, Retrospective Case Series [39]	**PFS** mean (SD)<br>20.0 (14.4) mo	**36-item Short Form Survey**<br>Improvement	**Blood BHB** (mmol/L) mean (SD) (n=12)<br>1.6 (1.1)		**Side Effect**<br>No SE reported<br>**Tolerability**<br>NA
van der Louw et al. 2018, Netherlands, Prospective Case Series [40]	**OS** from diagnosis mean (range)<br>13.8 (6.4- 18.7) mo	NA	NA	SD score for **weight for height**<br>Pt 1 Baseline +0.05; End of the study −0.35<br>Pt 2 Baseline −3.09; End of the study −0.30<br>Pt 3 Baseline +2.16; End of the study +2	**Side Effect**<br>CTCAE fatigue, vomiting, food refusal, constipation, inability to swallow<br>**Tolerability**<br>Well tolerated<br>The introduction of some ketogenic snacks improved the tolerance

Zapata Laguado et al. 2024, Colombia, Prospective Case Series [41]	NA	NA	NA	**Weight** (Kg) mean (range)<br>Baseline 66.2 (42-91); changes 5 pt loss weight, 24 pt maintained weight	**Side Effect**<br>Gastrointestinal symptoms grade I diarrhea (5 pt), vomiting (3 pt), constipation (2 pt)<br>**Tolerability**<br>NA
Artzi et al. 2017, Israel, Quasi-Experimental [17]	NA	NA	**Urine ketones** (mmol/L)<br>≥4 in all pt after 1 mo<br>**Cerebral Ketones** metabolism on MRS CKD group: AcAc and/or acetone were detected in 1 pt in the NAWM or lesion areas; acetone was detected in 1pt in lesional area. Control group: no ketone bodies in the brain	NA	**Side Effect**<br>NA<br>**Tolerability**<br>Well tolerated in 4/5 pt
Foppiani et al. 2021, Italy, Quasi-Experimental [42]	NA	NA	**Blood Pre-prandial Glycemia** (mg/dL) mean (SD)<br>Baseline 96 (11); 1 mo 93 (7) p=0.643<br>**Blood Pre-prandial ketonemia** (mmol/L) mean (SD)<br>Baseline 0.3 (0.1); 1 mo 1.7 (1.0) p=0.024*	**Weight** (Kg) mean (SD)<br>Baseline 71.8 (10.4); End of the study 68.3 (9.4) p=0.013*<br>**BMI** (kg/m2) mean (SD)<br>Baseline 22.9 (3.3); End of the study 21.8 (3.0) p=0.013*	**Side Effect**<br>Frequent mild hyperglycemia events (max value 115 mg/dl)<br>**Tolerability**<br>NA
Klein et al. 2020, USA, Quasi-Experimental [43]	**OS** from diagnosis mean (range)<br>Group 1: 21.8 (11–29.2) mo<br>Group 2: 25.4 (13.9–38.7) mo<br>**OS** after start of the diet mean (range)<br>Group 1: 20 (9.5–27) mo<br>Group 2: 12.8 (6.3–19.9) mo<br>**PFS** mean (range)<br>Group 1: 3.4 (1.6–7.1) mo<br>Group 2: 3.9 (0.6–9.1) mo	NA	**FPG** during diet (mg/dL) mean (range)<br>Group 1 85.9 (76.9-101.3); Group 2 92.17 (76,15-105,46)<br>**Blood BHB** during diet (mmol/L) mean (range)<br>Group 1 2.9 (2.3-3.6); Group 2 0.5 (0.2-1)<br>**Urine Ketones** during diet (mg/dL) mean (range)<br>Group 1 63.6 (58.6-68.2); Group 2 22.4 (4.7-37.9)	**Weight** (Kg)<br>All 6 pt treated for > 1 mo experienced weight loss, ranging from 4.9–8.1<br>**BMI** (kg/m2)<br>reduction range 1.9–2	**Side Effect**<br>TEAEs mild-transient weight loss and hunger (6 pt), nausea (3 pt), dizziness (2 pt), fatigue (1 pt), constipation (1 pt)<br>**Tolerability**<br>Well tolerated
Martin-McGill et al. 2018, UK, Quasi-Experimental [44]	NA	NA	**Urine Ketones** (mmol/L)<br>≥4 in all pt within 1 wk. 3 pt maintained ketosis during the study	**Weight** (Kg) mean (SD)<br>Baseline 85.6 (11.7); End of the study 84.6 (9.6) p=0.71<br>**BMI** (kg/m2) mean (SD)<br>Baseline 25.2 (23–29.6); End of the study 25.1 (23.4–28.4) p=0.75	**Side Effect**<br>Constipation (2 pt)<br>**Tolerability**<br>Well tolerated
Martin-McGill et al. 2020, UK, Quasi-Experimental [45]	**OS** mean (range) from surgery<br>MCT KD 59.3 (35.4-83.6) wk<br>MKD 52 (31.6-67.3) wk<br>**PFS** mean (range)<br>MCT KD 25.82 (14-44.4) wk	**EORTC QLQ-C30 (GHS)**<br>At the end of the study, GHS improved in the MKD (1pt) and worsened in the MCT KD (2pt)	**Urine Ketones** (mmol/L) in the First 6 wk<br>MCT KD (3 pt) ≥4; MKD (3 pt) ≥4<br>Urine Ketones (mmol/L) after 50 wk<br>MCT KD (3 pt) 4; MKD (2 pt) 8	**Weight** (Kg) mean (SD)<br>MCT KD (3 pt) Baseline 88.5 (11.3); At 12 mo 82.3 (1.3);<br>MKD (1 pt) Baseline 130.5; At 12 mo 96.6	**Side Effect**<br>MCTKD<br>CTCAE grade I hypokalemia (2 pt), hypernatremia (1 pt), hypocalcemia (1 pt), partial seizure (1 pt), diarrhoea (1 pt), nausea (1

	MKD 22.10 (5.1-64.3) wk	**Qualitative interviews**<br>At the end of the study, both groups reported to experiencing a 'fantastic quality of life'		**BMI** (kg/m2) median (range)<br>MCT KD (3 pt) Baseline 29.1 (25.1-33.3); At 12 mo 27.2 (27.1-27.2); MKD (1 pt) Baseline 35.8; At 12 mo 28.9	pt), vomiting (1 pt), dyspepsia (2 pt), constipation (1 pt)<br>MKD<br>CTCAE grade I vomiting (1 pt), dry mouth (1 pt), constipation (1 pt)<br>**Tolerability**<br>Not well tolerated
Porper et al. 2021, Israel, Quasi-Experimental [46]	**OS** median<br>New diagnosis: 21 mo<br>Recurrent: 8 mo<br>**PFS** median<br>New diagnosis: 10 mo<br>Recurrent: 4 mo	NA	**Blood Glucose** (mg/dL) mean<br>Baseline 99.8; During treatment 96.21 p=0.4<br>**Blood BHB** (mmol/L) mean<br>Baseline 0.09; During treatment 0.59 p=0.006*	**Weight** (Kg) mean<br>Baseline 85.1; During diet 84.4 p=0.4<br>**BMI** (kg/m2) mean<br>Baseline 29.48; During diet 29.16 p=0.3	**Side Effect**<br>CTCAE grade III in 2 pt nausea and asymptomatic hyperuricemia<br>CTCAE grade I-II in 12 pt anorexia (6 pt), nausea (5 pt), weight loss (1 pt), vomiting (3 pt), constipation (3 pt), diarrhea (1 pt), hiccups (1 pt), hypercholesterolemia (8 pt), seizures (1 pt)<br>**Tolerability**<br>Well tolerated
Rieger et al. 2014, Germany, Quasi-Experimental [47]	**OS** median (range) after start of the diet: 32 (6-86+) wk<br>PFS median (range)<br>5 (3-13) wk	NA	**Blood Glucose** (mg/dlL mean (SD)<br>Baseline 99 (21.8); During treatment 92 (9.1)<br>**Urine Ketones** (mmol/L)<br>Almost all pt maintained ketosis during the study	**Weight** (Kg) mean (SD)<br>Before diet 78.3(16.1); During diet 76.5(14.6) statistically significant weight loss of ~2.2% during the diet	**Side Effect**<br>No serious SE attributable to the diet. Few pt reported diarrhea, constipation, hunger, demand for glucose<br>**Tolerability**<br>Well tolerated
Santos et al. 2018, Brazil, Quasi-Experimental [48]	NA	NA	NA	**Weight** (Kg) median<br>Before diet 81.8; During diet 81.0<br>**BMI** (kg/m2) median<br>Before diet 29.69; During diet 29.16	**Side Effect**<br>No SE reported<br>Tolerability<br>Well tolerated
Schreck et al. 2021, USA, Quasi-Experimental [49]	NA	NA	**Blood Fasting glucose** (mg/dL) mean (SD)<br>Baseline 92.3 (10.3); End of the study 87.3 (11.3) p=0.057<br>**Urine Ketones** (mg/dL)<br>80% pt maintained ketosis ≥40 during the study<br>**Cerebral ketones** metabolism on MRS<br>Significant increases in BHB and acetone concentrations in both lesional (BHB p=0.011, acetone p=0.012) and contralateral (BHB p=0.031, acetone p=0.005) brain compared to baseline<br>Average ketonuria correlated with cerebral ketones in lesional (tumor) and contralateral brain (BHB Rs = 0.52, p = 0.05)	**Weight** (Kg) mean (SD)<br>Baseline 78.8 (16.7); End of the study 75.4 (15.5) p=<0.0001*<br>**BMI** (kg/m2) mean (SD)<br>Baseline 26.2 (5.6); End of the study 25.4 (5.4) p=<0.0001*	**Side Effect**<br>CTCAE grade II leukopenia (3 pt), nausea (2 pt), colitis (1 pt), diarrhea (1 pt), fatigue (1 pt), headache (1 pt), myalgias (1 pt), leukocytosis (1 pt), seizure (1 pt).<br>CTCAE grade III neutropenia (1 pt possibly related)<br>**Tolerability**<br>Well tolerated

van der Louw et al. 2019, Netherlands, Quasi-Experimental [50]	**OS** median (IQR) from diagnosis<br>12.8 (12.3–17.7) mo	**EORTC QLQ-C-30**<br>outcomes did not change essentially during the study	**Blood Glucose** (mmol/L) mean (SD)<br>CKD 4:1 4.7 (0.17); MCTKD 5.2 (0.70)<br>**Blood Ketones** (mmol/L) mean (SD)<br>9 patients reached adequate ketosis (>3mmol/L)<br>CKD 4:1 4.3 (1.20); MCTKD 2.9 (1.17)	**BMI** (kg/m2) median (IQR)<br>Baseline 25 (2.5); End of the study 24 (4.2)	**Side Effect**<br>CTCAE Grade I Constipation (7 pt), nausea/vomiting (2 pt), hypercholesterolemia (1 pt), hypoglycemia (1 pt), low carnitine (1 pt), diarrhea (1 pt)<br>CTCAE Grade II Hallucinations (1 pt), allergic reaction (1 pt), wound infection (1 pt)<br>Severe AEs: Seizures (3 pt), ambulatory issues (1 pt), recurrent wound infection (1 pt).<br>**Tolerability**<br>NA
Woodhouse et al. 2019, USA, Quasi-Experimental [18]	**2-year OS**<br>4 of 15 (26.7%)	NA	**Blood BHB** (mmol/L)<br>23 of the 29 pt >1	**BMI** (kg/m2) median change: –1.04<br>Decrease 25 pt (1 underweight pt); Increase 3 pt; No change 1 pt	**Side Effect**<br>No serious SE, no grade III or IV toxicities developed. Grade II constipation (1 pt)<br>**Tolerability**<br>Well tolerated
Voss et al. 2020, Germany, RCT [51]	**OS** median (95% CI)<br>MKD-IF group: 331 (124-538) days Control group: 291 (165-417) days (p=0.978)<br>**PFS** median (95% IC)<br>MKD-IF group: 75 (0-155) days<br>Control group: 91 (39-148) days (p=0.729)	NA	**Blood Glucose** (mg/dL) difference between baseline and day 6 mean (SD)<br>MKD-IF group - 11.2 (16); Control group 1.4 (11.2) p < .01<br>**Ketones** (mmol/L) difference between baseline and day 6 mean (SD)<br>MKD-IF group 1.9 (1.5); no change in the Control group p < .01	**Weight** (Kg)<br>mean (SD) difference between baseline and day 6 (kg)<br>MKD-IF group: -2.1 (1.8); Control group: -0.7 (1.4) p=0.008	**Side Effect**<br>Headache, nausea, seizure possible epileptic seizures with short-lasting aphasia in MKD-IF (4 pt); Control group (5 pt)<br>**Tolerability**<br>Well tolerated
Voss et al. 2022, Germany, RCT [52]	**OS** mean (95% CI)<br>MKD-IF group: 394 days (270–519) Control group: 374 days (274–473) (p =0.965)<br>**PFS** mean<br>MKD-IF group: 122 days (61–184) Control group: 111 days (73–149)<br>(p =0.845)<br>PFS mean in MKD-IF group patients with glucose levels on day 6 below the median (of 83.5 mg/l): 167 days (55-279) patients with glucose levels on day 6 above the median (of 83.5 mg/l): 52 days (34-70) (p=0.014).	**EORTC QLQ-C30 (GHS)**<br>no significant difference among MKD-IF group and control group during treatment or at later follow-up 1 mo later	NA	NA	**Side Effect**<br>NA<br>**Tolerability**<br>Well tolerated

Legend: AcAc acetoacetate; **AEs** adverse effects; **BHB** beta-hydroxy butyrate; **BMI** body max index; **CKD** classic ketogenic diet; **CTCAE** common terminology criteria of adverse events**; DVT** deep venous thrombosis; **EORTC-QLQ-C30** European Organization for Research and Treatment of Cancer Quality of Life Questionnaire Core 30; **FPG** Fasting plasma glucose; **GHS** Global Health Status; **GKI** glucose ketone index; **IQR** interquartile range; **IF** intermittent fasting; **MAD** Modified Atkins Diet; **MCT KD** medium chain triglyceride ketogenic diet; **MKD** modified ketogenic diet; **MO** months; **MRS** Magnetic resonance spectroscopy; **OS** overall survival; **NA** not available; **NAWM** normal appearing white matter; **PFS** progression free survival; **SD** standard deviation; **SE** side effects; **TEAE** treatment emergent adverse event; **WK** weeks.

Section and Topic	Item #	Checklist item	Location where item is reported
TITLE			
Title	1	Identify the report as a systematic review.	Pages 1-4
ABSTRACT			
Abstract	2	See the PRISMA 2020 for Abstracts checklist.	Done
INTRODUCTION			
Rationale	3	Describe the rationale for the review in the context of existing knowledge.	Pages 2-4
Objectives	4	Provide an explicit statement of the objective(s) or question(s) the review addresses.	Pages 3-4
METHODS			
Eligibility criteria	5	Specify the inclusion and exclusion criteria for the review and how studies were grouped for the syntheses.	Pages 3-5
Information sources	6	Specify all databases, registers, websites, organisations, reference lists and other sources searched or consulted to identify studies. Specify the date when each source was last searched or consulted.	Page 3-4
Search strategy	7	Present the full search strategies for all databases, registers and websites, including any filters and limits used.	Page 4, Supplementary Table 1
Selection process	8	Specify the methods used to decide whether a study met the inclusion criteria of the review, including how many reviewers screened each record and each report retrieved, whether they worked independently, and if applicable, details of automation tools used in the process.	Pages 4, 5, Supplementary Table 2
Data collection process	9	Specify the methods used to collect data from reports, including how many reviewers collected data from each report, whether they worked independently, any processes for obtaining or confirming data from study investigators, and if applicable, details of automation tools used in the process.	Pages 4, 5, Supplementary Table 2
Data items	10a	List and define all outcomes for which data were sought. Specify whether all results that were compatible with each outcome domain in each study were sought (e.g. for all measures, time points, analyses), and if not, the methods used to decide which results to collect.	Pages 4, 5, Supplementary Table 2
	10b	List and define all other variables for which data were sought (e.g. participant and intervention characteristics, funding sources). Describe any assumptions made about any missing or unclear information.	Pages 4, 5
Study risk of bias assessment	11	Specify the methods used to assess risk of bias in the included studies, including details of the tool(s) used, how many reviewers assessed each study and whether they worked independently, and if applicable, details of automation tools used in the process.	Page 5
Effect measures	12	Specify for each outcome the effect measure(s) (e.g. risk ratio, mean difference) used in the synthesis or presentation of results.	Pages 4, 5
Synthesis methods	13a	Describe the processes used to decide which studies were eligible for each synthesis (e.g. tabulating the study intervention characteristics and comparing against the planned groups for each synthesis (item #5)).	Pages 4, 5
	13b	Describe any methods required to prepare the data for presentation or synthesis, such as handling of missing summary statistics, or data conversions.	Pages 4, 5
	13c	Describe any methods used to tabulate or visually display results of individual studies and syntheses.	Pages 4, 5

Section and Topic	Item #	Checklist item	Location where item is reported
	13d	Describe any methods used to synthesize results and provide a rationale for the choice(s). If meta-analysis was performed, describe the model(s), method(s) to identify the presence and extent of statistical heterogeneity, and software package(s) used.	Pages 4, 5
	13e	Describe any methods used to explore possible causes of heterogeneity among study results (e.g. subgroup analysis, meta-regression).	Pages 4, 5, supplementary material
	13f	Describe any sensitivity analyses conducted to assess robustness of the synthesized results.	
Reporting bias assessment	14	Describe any methods used to assess risk of bias due to missing results in a synthesis (arising from reporting biases).	Pages 5
Certainty assessment	15	Describe any methods used to assess certainty (or confidence) in the body of evidence for an outcome.	
RESULTS			
Study selection	16a	Describe the results of the search and selection process, from the number of records identified in the search to the number of studies included in the review, ideally using a flow diagram.	Page 5-7, Figure 1
	16b	Cite studies that might appear to meet the inclusion criteria, but which were excluded, and explain why they were excluded.	Figure 1, Supplementary Table 3
Study characteristics	17	Cite each included study and present its characteristics.	Pages 6-10, Tables 3 and 4
Risk of bias in studies	18	Present assessments of risk of bias for each included study.	Pages 19,20, Table 4
Results of individual studies	19	For all outcomes, present, for each study: (a) summary statistics for each group (where appropriate) and (b) an effect estimate and its precision (e.g. confidence/credible interval), ideally using structured tables or plots.	Tables 3 and 4, Page 20-23
Results of syntheses	20a	For each synthesis, briefly summarise the characteristics and risk of bias among contributing studies.	Pages 20-23, Tables 3 and 4
	20b	Present results of all statistical syntheses conducted. If meta-analysis was done, present for each the summary estimate and its precision (e.g. confidence/credible interval) and measures of statistical heterogeneity. If comparing groups, describe the direction of the effect.	Pages 20-23, Tables 3 and 4
	20c	Present results of all investigations of possible causes of heterogeneity among study results.	Pages 20-23, Tables 3 and 4
	20d	Present results of all sensitivity analyses conducted to assess the robustness of the synthesized results.	
Reporting biases	21	Present assessments of risk of bias due to missing results (arising from reporting biases) for each synthesis assessed.	Pages 19,20, Table 4

Section and Topic	Item #	Checklist item	Location where item is reported
Certainty of evidence	22	Present assessments of certainty (or confidence) in the body of evidence for each outcome assessed.	
DISCUSSION			
Discussion	23a	Provide a general interpretation of the results in the context of other evidence.	Pages 23-26
	23b	Discuss any limitations of the evidence included in the review.	Pages 23-26
	23c	Discuss any limitations of the review processes used.	Page 26
	23d	Discuss implications of the results for practice, policy, and future research.	Page 26
OTHER INFORMATION			
Registration and protocol	24a	Provide registration information for the review, including register name and registration number, or state that the review was not registered.	Page 4
	24b	Indicate where the review protocol can be accessed, or state that a protocol was not prepared.	Page 4
	24c	Describe and explain any amendments to information provided at registration or in the protocol.	
Support	25	Describe sources of financial or non-financial support for the review, and the role of the funders or sponsors in the review.	None
Competing interests	26	Declare any competing interests of review authors.	None
Availability of data, code and other materials	27	Report which of the following are publicly available and where they can be found: template data collection forms; data extracted from included studies; data used for all analyses; analytic code; any other materials used in the review.	Seach strategy: Supplementary Table 1; Protocol features: Supplementary Table 2; Excluded studies: Supplementary Table 3; Data extracted from included studies: Tables 3 and 4, Supplementary table 4

From: Page MJ, McKenzie JE, Bossuyt PM, Boutron I, Hoffmann TC, Mulrow CD, et al. The PRISMA 2020 statement: an updated guideline for reporting systematic reviews. BMJ 2021;372:n71. doi: 10.1136/bmj.n71. This work is licensed under CC BY 4.0. To view a copy of this license, visit https://creativecommons.org/licenses/by/4.0/
